# Stereotactic Body Radiation Therapy (SBRT) for Unresectable Pancreatic Carcinoma 

**DOI:** 10.3390/cancers2031565

**Published:** 2010-08-09

**Authors:** Michael C. Stauder, Robert C. Miller

**Affiliations:** Department of Radiation Oncology, Mayo Clinic, Rochester, MN, USA; E-Mail: stauder.michael@mayo.edu

**Keywords:** stereotactic radiotherapy, radiotherapy, pancreatic neoplasms

## Abstract

Survival in patients with unresectable pancreatic carcinoma is poor. Studies by Mayo Clinic and the Gastrointestinal Tumor Study Group (GITSG) have established combined modality treatment with chemotherapy and radiation as the standard of care. Use of gemcitabine-based chemotherapy alone has also been shown to provide a benefit, but 5‑year overall survival still remains less than 5%. Conventional radiotherapy is traditionally delivered over a six week period and high toxicity is seen with the concomitant use of chemotherapy. In contrast, SBRT can be delivered in 3–5 days and, when used as a component of combined modality therapy with gemcitabine, disruption to the timely delivery of chemotherapy is minimal. Early single-institution reports of SBRT for unresectable pancreatic carcinoma demonstrate excellent local control with acceptable toxicity. Use of SBRT in unresectable pancreatic carcinoma warrants further investigation in order to improve the survival of patients with historically poor outcomes.

## 1. Introduction

Pancreatic carcinoma is an uncommon cancer, representing only 3% of all malignancies and 14% of gastrointestinal malignancies. Despite this, it is the fourth leading cause of cancer death in the United States. According to the American Cancer Society 2009 statistics, pancreatic carcinoma has a projected incidence of 42,470 in the United States and will cause an estimated 35,240 deaths [1]. The estimated 5-year overall survival for patients with pancreatic carcinoma is 5% or less, and has not changed significantly over the last 30 years. Therefore, investigations into methods of improving survival in pancreatic carcinoma continue to be pursued. Surgical resection can prolong survival in select cases and is the only treatment modality offering a chance of disease eradication. Unfortunately, only about 10–15% of patients are candidates for potentially curative resection at diagnosis and, as a result, outcomes have traditionally been poor. Even in node-negative patients undergoing optimal pancreaticoduodenectomy, 5-year survival is only 24% [2]. In addition, both local and systemic recurrences are common after surgical resection [3]. Therefore, new investigations into more effective treatments are warranted.

## 2. Radiation Therapy for Locally Advanced Pancreatic Carcinoma

There are many limitations to the use of radiation therapy alone as definitive therapy for unresectable pancreatic carcinoma. For example, external beam radiotherapy has traditionally been limited by the radiation tolerance of adjacent tissue, primarily the duodenum. When given as conventionally fractionated therapy, accepted limits for maximum radiation dose to the duodenum are thought to be about 50 Gray (Gy) to one-third of the organ or 40 Gy to the entire organ [4]. At these dose levels, it is estimated that there is a 5% probability of treatment-related complications within five years of treatment. More recently, dose-volume constraint recommendations have been redefined with the recent publication of Quantitative Analyses of Normal Tissue Effects in the Clinic (QUANTEC). The data analyzed as part of this publication recommends that only 195 cm^3^ of small bowel receive >45 Gy [5]. However, biologically effective doses in excess of 70 Gy are often necessary to achieve a high probability of tumor control [6]. 

Conventionally fractionated radiation takes advantage of the deficiencies in DNA-damage repair inherent to many tumor cells. This results in making ionizing radiation more lethal to malignant cells than to those of normal tissue. At higher doses per fraction, emerging data suggests that in addition to direct cytotoxicity, a different mechanism involving microvascular damage begins to have a substantial effect on tumor cell kill [7,8]. Technological advances in stereotactic body radiation (SBRT) have built on the well-established principles of intracranial radiosurgery to allow delivery of a single or limited number of high dose radiation fractions to extracranial tumor targets [9]. This enables the delivery of ablative doses to the tumor and immediately adjacent tissues that is analogous to a surgical resection [10]. In fact, the use of SBRT has become an alternative to resection when involvement of critical structure precludes a traditional surgical approach. Recent trials have demonstrated the tolerability and efficacy of SBRT for treatment of early-stage lung cancer and liver metastases. For example, the published results for SBRT in early-stage non-small cell lung cancer have shown encouraging outcomes with reports of local control rates of 80–100% [11,12,13,14,15].

As the dose per fraction increases with the use of SBRT, the ability to delineate between tumor and organs at risk becomes essential in order to adequately target disease, but also to avoid potential complications of high doses of radiation on normal tissues. In most reports, the planning target volume (PTV) represents the gross tumor volume (GTV) as visible on pre-treatment imaging studies plus a margin to account for tumor displacement due to respiration (internal target volume or ITV) and patient set up uncertainty. Various techniques are also utilized to aid in tumor localization. For example, treatment planning is frequently done with respiratory-correlated cone-beam computed tomography (4D-CT) as described by D’Souza *et al*. [16], with abdominal compression to limit the respiratory-associated movement of tumor. Often, gold fiducial markers are implanted within the patient’s tumor to be used during the course of treatment to actively track tumor movement. Image guided techniques such as cone-beam CT, as described by Pouliot *et al*. [17] and Chen *et al*. [18] can also be used to confirm tumor position prior to delivering each fraction of radiation.

## 3. Combined Modality Therapy for Locally Advanced Pancreatic Carcinoma

The use of 5-flurouracil (5-FU) based chemotherapy, in combination with external beam radiation therapy, has demonstrated a survival advantage over the use of radiation therapy alone [19,20,21]. Recent reports have established the superiority of gemcitabine-based chemotherapy over 5-FU when used alone or in combination with radiation therapy. In patients with symptomatic advanced pancreas cancer, gemcitabine, when given alone as a weekly regimen, was shown to have improved symptom control and a modest survival benefit compared to 5-FU [22]. *In vitro* studies also support the fact that gemcitabine acts as a potent radiosensitizer [23,24] and when used in combination with radiation therapy, survival outcomes are improved. In a Phase III randomized study of gemcitabine in combination with radiation therapy *versus* gemcitabine alone, Loehrer *et al*. reports a statistically significantly improvement in overall survival with the use of combined modality therapy consisting of gemcitabine and radiation therapy [25]. Patients enrolled in this trial were randomized to either gemcitabine (1000 mg/m^2^) alone given weekly × 3 every 4 weeks for 7 cycles or radiation therapy 50.4 Gy in 28 fractions along with gemcitabine (600 mg/m^2^) weekly × 6 followed by 5 cycles of gemcitabine alone (1000 mg/m^2^) given weekly × 3 every 4 weeks. In patients receiving radiation therapy, an improvement in median overall survival of 11.0 months was seen compared to 9.2 months in patients receiving gemcitabine alone. The objective response rate and progression free survival were also improved in patients randomized to receive radiation therapy.

Although most patients with pancreatic cancer eventually die of metastatic disease, data suggests that local control of disease is important, as well. A study from Taiwan, using 50.4 to 61.2 Gy with concomitant chemoradiotherapy as treatment for unresectable pancreatic carcinoma, reported that 6 of 18 patients (33%) had local progression as a component of their treatment failure [26]. Also, a report from MD Anderson Cancer Center on 323 patients who received induction chemotherapy and conventional radiotherapy, reports a 46% rate of local progression with or without distant progression. Among these patients, a total of 56 (38%) had local progression alone [27]. This emphasizes the need for more effective local therapy which will likely require novel combinations of systemically active therapies combined with radiotherapy.

## 4. Results of Stereotactic Body Radiation Therapy for Unresectable Pancreatic Carcinoma

Several trials have evaluated the feasibility, toxicity and tumor response of SBRT for unresectable, locally advanced pancreatic carcinoma (Table 1). One of the earliest Phase II studies involving SBRT in patients with non-resectable tumors due to invasion of vessels or other adjacent structures was performed in Denmark. In this trial, a dose of 45 Gy in 3 fractions was delivered over a period of 5–10 days to the treatment isocenter. Also, the dose at the margin of the tumor corresponded to the 67% isodose line or 30 Gy. The treatment volumes as described in this study were much larger than more contemporary trials looking at SBRT as a component of management for pancreatic carcinoma. The patients in this series had poor outcomes, suggesting that treating such a large volume may be detrimental to durable tumor control. A median survival of 5.7 months was seen in this study and, of the 22 patient evaluated, only 5% were alive at 1 year post treatment. A median time to progression of 4.8 months was seen with a one year progression free survival of 9%. Additionally, only one patient was free from disease progression at 18 months [28]. 

**Table 1:** SBRT for locally advanced pancreatic carcinoma.

Study	Fractionation	Median follow-up (months)	Local control (%)	Median survival (months)	Grade ≥ grade 3 toxicity (%)	
					Acute	Late
Chang *et al*. [30]	25 Gy × 1	12	84	11.9 *	1	9
Hoyer *et al*. [28]	45 Gy × 3^†^	6	57	5.4	78	33
Mahadevan *et al*. [32]	8–12 Gy × 3	24	78	14.3	8	6
Polistina *et al*. [38]	10 Gy × 3	9	83	10.6	0	0
Rwigema *et al*. [31]	18–24 Gy × 1^††^	6	77	10.3	4	0

* Calculated from time of diagnosis. †Dose to ICRU reference point; †† Various doses delivered: 24 Gy (61%), 22 Gy (18%), 25 Gy (7%), 20 Gy (6%), 18 Gy (3%).

As seen with conventional radiation therapy, patient outcomes are improved when gemcitabine is added to SBRT. Investigators from Stanford University have reported the results of a single institution Phase 2 trial of gemcitabine and single-fraction SBRT in locally advanced, nonmetastatic, pancreatic adenocarcinoma. A total of 16 patients, who were treated with gemcitabine and SBRT to 25 Gy in a single fraction delivered two weeks after completion of the first cycle, are detailed in this study. A median survival of 11.4 months was seen with a 1 year overall survival of 50% and an estimated 2 year survival of 18% [29]. A cumulative report of all 77 patients treated with single fraction SBRT reports similar outcomes [30]. The rates of freedom from local progression at 6 months and 12 months were 91% and 84%, respectively. Of the patients with progressive disease, only 13% had local failure as a component of their disease progression. The median survival of all patients treated with single fraction SBRT, as calculated from the time of diagnosis, was 11.9 months.

Other institutions have published data on the use of SBRT in locally advanced pancreatic carcinoma as well. Rwigema *et al*. describes the University of Pittsburgh experience in a heterogeneous group of 70 patients, including some with metastatic disease, who received SBRT as part of their management [31]. Most patients in this study received a prescription dose of 24 Gy delivered to the 80% isodose line (marginal dose of 19.2 Gy) in a single fraction. A total of 40 patients (56%) described in this study had locally advanced, unresectable disease. Another 11 patients (16%) were treated for local recurrence after previous external beam radiation therapy (median 45 Gy), and 12 patients (17%) were given SBRT in the adjuvant setting for positive or close margins. Despite this heterogeneity, good local control and overall survival was seen in the entire patient population. In fact, in a select group of patients with tumors <15 mL in size, freedom from local progression was achieved in 77.3% at a median follow up of 12.7 months. Among all patients, a median survival of 10.3 months was reported with a 6 and 12-month overall survival rate of 65.3% and 41 % respectively. 

A study by Polistina *et al*. has reported the experience of 23 patients with locally advanced, unresectable pancreatic carcinoma given 30 Gy SBRT in 3 consecutive daily fractions. All patients also received gemcitabine chemotherapy for 6 weekly cycles after SBRT. In this group of patients, the overall local control rate was 82% at 6 month follow-up visits. In addition, 5 patients (22%) had their disease become resectable after treatment.

Recently published data by Mahadevan *et al*. on combined modality therapy from Beth Israel Deaconess Medical Center details a different approach to treatment [32]. In the 36 patients evaluated, a radiation dose of 24 Gy, 30 Gy, or 36 Gy in 3 fractions was prescribed based on the location of tumor in relation to the duodenum. If the tumor abutted one third or more of the circumference of the duodenum, a dose of 24 Gy was delivered. If the tumor touched the duodenum in only one area or the space between the tumor and bowel was <3 mm, a dose of 30 Gy was delivered. A higher dose of 36 Gy was given if tumor was found to be ≥3 mm from the duodenum. All patients received gemcitabine chemotherapy weekly × 3, every 28 days for 6 cycles starting one month after SBRT. However, despite the location-based dosing, most patients in this series (69%) received a total of 30 Gy in 3 fractions. A median survival of 14.3 months was seen with a local control rate of 78% at a median follow-up duration of 24 months (range 12–33). In this study, progression-free survival as determined by CA 19-9 was 7.9 months and 9.6 months if determined by CT scan.

## 5. Risks of Toxicity

One of the prevailing limitations to more widespread acceptance of SBRT for locally advanced pancreatic carcinoma has been the toxicities associated with treatment, especially when combined with concomitant chemotherapy. The early GITSG reports did not report late toxicity, but many instances of unacceptable toxicity were seen in other reports of combined modality treatment with gemcitabine and conventionally fractionated radiation therapy. Patients enrolled in a Phase I dose escalation study described by McGinn *et al*. were administered gemcitabine as a 30-minute intravenous infusion at a dose of 1000 mg/m^2^ on days 1, 8, and 15 of a 28-day cycle together with external beam radiation therapy. Due to the toxicity of treatment, the authors recommend a dose no greater than 36 Gy in 2.4-Gy fractions when gemcitabine is given concomitantly [33]. Using this treatment regimen, sixteen of seventy-four patients (22%) had ≥Grade 3 gastrointestinal toxicity. An 11% rate of late ≥grade 3 toxicity, including 6 episodes of upper GI bleeding, was reported. In addition, a 69% incidence of acute grade 1–2 gastrointestinal toxicity was seen. The acute toxicities primarily consisted of nausea, vomiting and diarrhea, but there were also 2 upper GI bleeds as well as a duodenal fistula and a partial small bowel obstruction reported. Other significant toxicities seen in these patients included a colon stricture and a choledochoduodenal fistula. In addition, one death was reported from a bleeding duodenal ulcer [34]. 

In early SBRT trials, a dose of 45 Gy in 3 fractions delivered to the pancreas, without the use of chemotherapy, was deemed to be too toxic. A probable contributing factor to the toxicity seen in this series is that a large volume was treated to moderately high doses, as the PTV (median 136 cm^3^) consisted of gross tumor with surrounding edema, plus an isotropic margin of 5 mm in the transverse direction, and 10 mm in the cranio-caudal direction. Many patients receiving this dose reported a decrease in performance status, increased nausea, and more pain after treatment compared to baseline. In this trial, 5 of the 12 patients (42%) evaluated at ≥3 months suffered from severe mucositis or ulceration of the stomach or duodenum. In addition, one patient experienced a non-fatal perforated ulcer in the stomach. All of these patients had part of the stomach or duodenum receiving at least 67% of isocenter prescription dose, or 30 Gy in 3 Fx [28]. 

As reported by de Lange *et al*. similarly high rates of toxicity were seen in patients with locally advanced pancreatic carcinoma treated with concomitant gemcitabine administered at a dose of 300 mg/m^2^ 2–4 hours prior to hypofractionated radiotherapy (three weekly fractions of 8 Gy). This was followed by full dose gemcitabine (1000 mg/m^2^) given weekly × 3, every 28 days, for a median of 13 cycles. In this series of 24 patients, the authors report a 37.5% incidence of ulceration in the stomach (9 patients) with 5 of these having associated bleeding. Two deaths were reported, including one from a bleeding ulcer and another from the development of an aortoduodenal fistula [35]. 

The initial reports on the Stanford 25 Gy in 1 Fx regimen reported only mild acute gastrointestinal toxicity consisting of 2 cases of grade 2 and 1 case of grade 3 toxicity. Late toxicity, however, was much more common in reports of the Phase II component, as five ulcers (grade 2), one duodenal stenosis (grade 3), and one duodenal perforation (grade 4) were seen [29]. The cumulative experience of all patients at Stanford receiving a single fraction of 25 Gy by Chang *et al*. show that the grade ≥2 acute and late toxicity rates at 6 and 12-months are 17% and 28%, respectively [30]. In a recent dosimetric analysis of these patients, additional data is provided. A median time to toxicity of 6.3 months was reported with a significant decrease in duodenal toxicity seen on univariate analysis when the maximum dose to the duodenum was kept below 23 Gy [36]. A similar decrease in toxicity was seen when the volume of duodenum receiving 15 Gy, and 20 Gy were kept below the threshold of 9.1 cm^3^ and 3.3 cm^3^, respectively. Despite the higher rates of serious toxicity in early SBRT reports, compared to conventional chemoradiotherapy for pancreatic carcinoma, the overall toxicity risk of SBRT is acceptable given the high probability of local control. In comparison, a recent RTOG randomized controlled Phase III trial [37], evaluating the role of adjuvant radiation therapy in combination with either gemcitabine or 5-FU, reported a 58% incidence of grade 3 or higher toxicity in the group receiving concomitant gemcitabine.

The study by Mahadevan *et al*. [32] reports less toxicity when SBRT is delivered in three fractions compared to a single, high dose fraction. Of the 36 patients reported in this recent publication, 69% (25 patients) received a prescribed radiation dose of 30 Gy and all but 5 patients received gemcitabine after SBRT. With a median follow-up of 24 months, a total of 9 patients developed grade 2 nausea (25%) with 4 of these patients experiencing persistent nausea. There were also 3 patients (8%) who reported grade 3 toxicities. Hospitalization for control of cramping and vomiting was needed in 2 patients and an IVC thrombosis was seen in a third. In addition, late toxicity (≥3 months) occurred in2 patients (5.5%), both of which developed gastrointestinal bleeding requiring transfusion. 

Two other recent studies by Rwigema *et al*. [31], as well as an Italian study by Polistina *et al*. [38], report a low incidence of serious toxicity. In the University of Pittsburgh series, a total of three acute grade 3 toxicities were seen, consisting of one event each of nausea, abdominal pain, and gastroparesis. No late ≥ grade 2 toxicities were reported. The Italian study is remarkable for the lack of any grade 2 or higher toxicities. A summary of the incidence of ≥grade 3 toxicity for all published SBRT trials is presented in Table 1.

As reported in the above trials, toxicity rates after SBRT for pancreatic tumors compare favorably to a more protracted course of treatment, as long as dose thresholds for critical structures are respected. The recent QUANTEC data suggests that the small-bowel volume receiving >12.5 Gy in a single fraction should be kept to <30 cc with avoidance of circumferential coverage above that dose. The QUANTEC authors also provide a more conservative threshold such that for a three to five fraction regimen, the maximum point dose should be <30 Gy [5].

## 6. Biological Considerations of SBRT

The rationale for a multiple fraction regimen is based on putative advantages of fractionation, including reoxygenation of otherwise radioresistant hypoxic tumor and redistribution of tumor clonogens into a sensitive phase of the cell cycle. However, a conventional course of radiation is given over a number of weeks which can allow for interfraction repair of cellular radiation damage or lengthen any interruption of systemic therapy. Either of these factors could potentially compromise the effectiveness of therapy. In order to decrease the chance of significant interfraction repair, time committed to treatment and costs to the patient, hypofractionated courses of treatments are now commonly used. 

The dose of 36 Gy delivered in the Phase I dose-escalation trial of concomitant chemoradiotherapy described by McGinn *et al*. is equivalent to a biologically effective dose (BED) of 55 when calculated using an alpha/beta ratio of 3 for late responding tissues [33]. Similarly, a commonly prescribed dose for pancreatic carcinoma of 50.4 Gy in 28 fractions corresponds to a BED (Gy 3) of 62.5. In contrast, the Stanford University SBRT dose of 25 Gy in a single fraction and the Danish dose of 45 Gy in 3 fractions correspond to a BED (Gy 3) of 233 and 268, respectively. Radiobiologic principles support the fact that, at a given dose level, increasing the number of fractions can decrease the late effects of radiation on normal tissues. Given the excessive late duodenal toxicity seen after SBRT delivered at these doses, it would be justified to further increase the number of treatment fractions in order to decrease the BED and consequently, the probability of late duodenal toxicity. Therefore, clinical trials or other investigations looking at a more protracted SBRT, to deliver an equivalent or greater tumoricidal dose, but allow for decreased BED in regards to late normal tissue toxicity, need to be developed. 

## 7. Conclusions

Survival is poor in patients diagnosed with pancreatic carcinoma due to the lack of adequate treatment options. Only 15–20% of patients present with resectable disease, and even with adequate surgical resection, recurrence is common. Concurrent chemoradiotherapy is the standard of care at the present time, but conventionally fractionated radiation therapy can take 5–6 weeks to deliver. SBRT is a promising alternative to conventional radiation therapy in that local control is excellent and disruption to systemic therapy is minimal. The fact remains, however, that distant metastatic disease is common and toxicity of treatment is generally high. In order to address these issues, new investigations combining the use of a more protracted course of SBRT with chemotherapy need to be performed in the setting of well designed and conducted clinical trials.
